# Proceedings of International Conference on “Prescription for Wellness: Working Across Boundaries” held at Lahore Pakistan (August 2-3, 2019)

**DOI:** 10.12669/pjms.35.6.1655

**Published:** 2019

**Authors:** Shaukat Ali Jawaid, Nazish Imran

**Affiliations:** 1 Shaukat Ali Jawaid Chief Editor, Pakistan Journal of Medical Sciences; 2 Nazish Imran, FRC Psych. Dept. of Family & Child Psychiatry, King Edward Medical University, Lahore - Pakistan

Lahore: Department of Psychiatry and Behavioural Sciences at Fatima Memorial Hospital in collaboration with a number of other institutions including NUR International University, KEMU and Pakistan Association of Social Psychiatry organized an International Psychiatric Conference on “Prescription for Wellness: Working across Boundaries” held here from 2-3^rd^ August 2019. Prof. Khalid Masood Gondal Senior Vice President of College of Physicians & Surgeons Pakistan who is also Vice Chancellor of King Edward Medical University was the chief guest on this occasion. He made an announcement that CPSP will soon start Fellowship in Child Psychiatry. Candidates for this programme will be inducted in January 2020. Earlier Prof. Mowadat H.Rana had pointed out that all preliminary work including paper work and the MCQs etc., was completed over a year ago and if some officials of CPSP were telling him something else, it was wrong.


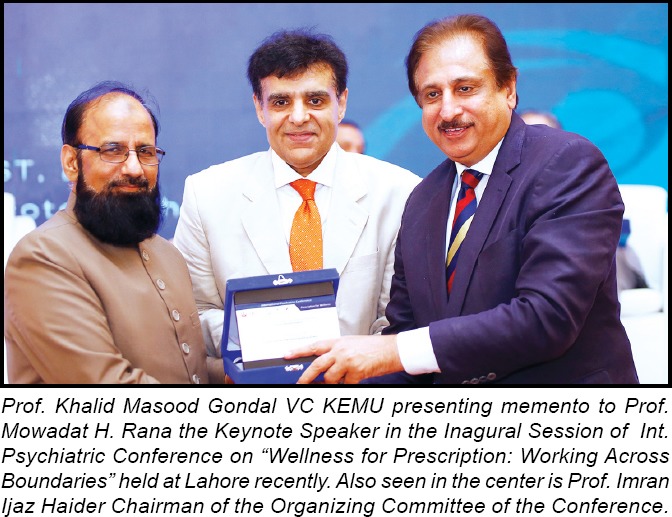


Prof.Khalid Masood Gondal congratulated the organizers for hosting a wonderful academic event and the high quality of the scientific programme including workshops on a wide variety of important topics. Mental Health and emotional well-being, he further stated, is vital part of a healthy and fulfilling life. At present mental ill health and psychological distress are globally receiving more recognition and there were numerous awareness and anti-stigma campaigns. It is necessary in our part of the world in particular so that misconceptions about mental illness, psychiatry and psychological interventions are removed. Often the sufferers are told to get over it or to strengthen themselves mentally and emotionally. In more severe cases people are labeled as mad and are ostracized. . Families are usually ashamed of such sufferers, and often will seek help from faith healers rather than medical professionals. The quality of life of such patients and their families is vastly compromised, and this is added to by the lack of supportive services and mental health infrastructure. Depression is considered as the “common cold” of psychiatry. WHO has noted that it is one of the leading causes of disability worldwide, that it is on the rise and that depression-related suicide is the 2^nd^ leading cause of death for people aged 19 – 25 Years. Depression is perhaps one of the most common diagnoses locally as well. These findings make it clear that quality mental health services are urgently required, with professionals being practiced in clinical and research skills.

People should be advised to consult qualified people by creating awareness as only qualified mental health professionals can treat them. We need quality mental healthcare services and find indigenous solutions to our problems. CPSP and universities have to play a leadership role.

**Prof. Mowadat H. Rana** in his thematic talk on the theme of the conference said that this was a unique conference. In the past most conferences were related to diseases but this one has given preference to Wellness and Happiness of the Healthcare professionals as well as the patients. Drugs, he recalled will never cure disability and distress. Make sure that the interventions we practice bring out the patient’s happiness, joy, comforts and their symptoms are going to be cured. We should be different from other doctors who just sit down and write prescriptions. We need to make a PLAN-what is the illness? The relationship of wellness and Health should be looked into and this plan should consist of evidence based wellness. We as mental healthcare professionals should promote wellness. He invited the pharma industry as well to come and invest in patient’s happiness and wellness which comes through happiness and health. If someone is diseased and still claims to be healthy he is suffering from manic episode. We must remember that happiness comes first and then health follows. Happiness reduces morbidity and the outcome is also different. Investment in happiness of patients will also increase efficacy of the medicines, he remarked. It is through promise of wellness that we can realize the full potential of a human being. It will also increase productivity, will result in better citizens. We Pakistanis are a very resilience nation and time has proved it time and again. We have always come out of traumatic growth as we believe in Allostasis- those who think ahead of time and foresee the problems, difficulties ahead.

Prof. Mowadat Rana further stated that we need a National Action Plan, personal commitment to health and happiness. He also highlighted the importance of spirituality stating that those patients who have belief have much more positive feelings and respond to treatment better. We must ensure that the patients are themselves committed to their health and well-being. Life style changes, Exercise and Nutrition programmes play a vital role in wellbeing. We need to infuse spirituality in the patients to ensure better prognosis. We should all show commitment to concept of Health, *Amar Bill Maroof* and *Naheen anal Munkar* should be involved as concept of Health, he remarked.

**Prof.Khalid Mufti** also highlighted the importance of the concept of Wellness and Spirituality. **Prof. Waqar Azeem** who had specially come from Qatar along with a team in his address commended the efforts of the organizers. He was delighted to see that the young psychologists, residents and Trainees were running the workshops. He specially referred to the session The Psyche Lounge which was moderated by two medical students. All this shows that the future of psychiatry is bright in Pakistan. **Prof. Mazhar Malik** pointed out that child psychiatry, substance abuse, psychosocial issues need to be highlighted and we must work in these neglected areas. **Prof. Unaiza Niaz** was delighted to note that this conference gave a refreshing look, the programme was very well planned and well designed, professionally organized and on the whole it had lot of innovations and the disciplined smooth working needs to be commended. In today’s modern family values, morality has been forgotten. The scientific programme provided numerous refreshing concepts. In the good old days, we the Asians, Muslim culture in particular had lot of moral values. We used to take nutritious food, relax and do regular exercise. We need to do something to improve child behaviour, give importance to women mental health as it will result in happy family and happy children.

**Prof.Rizwan Taj** referred to the out of control population and population under sixteen years of age need special attention. We need to create awareness and inculcate happiness among them. To achieve all that we need more academic events like this, he remarked. **Dr. Ajmal Kazmi** commended the team work witnessed during the conference. We the mental healthcare professionals, he opined, are facing many challenges.

Earlier **Prof. Imran Ijaz Haider** chairman of the organizing committee in his welcome address opined that let us think about positive things. Do not become Boss to the patient as most of us usually do not see beyond that. Let us remember that these patients are human beings as well and they need to be treated with respect and dignity apart from prescription of drugs. At present, mental health is an under-developed field in Pakistan as compared to more developed countries. This is in part because of socio-cultural stigma towards mental illness and treatment, as well as limited research output and lack of resources.

**Workshops on “Happiness for Healthcare Professionals”**

**Prof. Mowadat Rana** was the facilitator for this workshop which was jointly chaired by Prof. Khalid Mufti, Dr. Bushra, Prof.Aftab Asif and Dr. Burhan Mustafa. He pointed out that patients suffer from different categories which include Disease; Pill and they also suffer from the Doctors as well. These days’ doctors have become unhappy themselves and it is adding burden to the patients. It is the hurt people who see doctors to seek help and they get further hurt. It is a cycle and we need to break this vicious cycle, he remarked. We can start from some point to break this cycle. However, it is beyond the domain of health professionals in which people live. Doctors need to bring happiness to themselves first. He also referred to the validated index of happiness and then stated that there are some islands within the hospitals. Referring to Surah Al-Assar in the Holy Quran, Dr. Mowadat Rana said that it carries very important message. It lays emphasis on Truth, Patience and Perseverance. It further states that those who have Faith do Righteous Deeds. Assar is the time in the day to reflect and think about treatments, interventions for wellness. We must see what we have done so far during the day. What should one do better and correct the mistakes made so far. It is time for action for audit, reflect and make corrections. It also shows that there is still lot of time left during the day. We all sitting here, he went on to say, are in the age group where we all needs to do self audit.

Continuing Prof. Mowadat Rana said that there is a warning from God Almighty to reduce our loss. We are not happy. All new children enter in the world full of sadness, gloom. They all cry when they enter this world at the time of birth. Happiness, Dr. Mowadat Rana stated does not come, we have to find it and it is also expensive. There are five antidotes in this Surah Al-Assar and all those who understand it, their loss will be reduced. All these are measurable, tangible and doable. As stated by Hazrat Ali, our sources of pain and pleasure are the same. Sources of our wellness are potential sources of happiness. We are not happy with our relatives, parents, sibling, friends and neighbours and we need to find out a technique to turn this around. Diamond and Coal are found in the same place. We have a triangle of Mind, Body and Soul. Some people remain in their body and others use their Mind only. Body refers the case to others those living in the realm of Mind. Let us not get lost in Mind as well, he warned. There is a Soul also. All these three should live in harmony and Peace. When they live peacefully the outcome is happiness. We need to balance it. Within the same triangle there is another triangle i.e. I, God and Universe. Peaceful co-existence of all these is extremely essential. When we remember God, the chances of happiness are great. The triad required for happiness of healthcare professionals consists of Knowledge, Education and Skills. It is the combination of all these three which matters. Sorrows and Happiness are all part of life. What we need is Patience, live in it and wait for happiness, wait for sadness. Once you wait for happiness, it will come.

Giving details of the Happiness Index he mentioned Intent, Health, Freedom, and Conditions of life, Work, Voluntary and Number of loving relationship. On the other hand, there is Disease, Distress and Disability, Pain, Shame, Guilt and Vengeance and they all destroy happiness. Drugs are essential for disease but remember drugs won’t treat distress and disability. There are other ways to correct all this. The No.1 cause of breakdown of marriages these days are Tit for Tat approach. We lack patience. We do not know how to generate happiness. Alignment of ITSA is equal to Happiness wherein I stand for Intent, T stands for Thoughts, S for speech and A for Actions. We need to synchronize all these. Your Intent should be thought. Psychological pain is still more dangerous. Intentions are within one’s control. One can control one’s intentions. It should not be dependent on others actions, reactivity. It is better to use the frontal lobe. Do analysis, synthesis before reaction using frontal lobe. Respond but do not React. Stop reactions. Search guidance, help. Let emotions settle down and then respond, he added.

During the interactive discussions, it was emphasized that one should discuss with experts and make things available for discussion. Patients do not come to the doctors to get prescription of drugs but the come to discuss their problems. When this vicious cycle will stop, it will stop hurting people. Halal and Exercise restores confidence. Always opt for peer reviewed interventions. Patients need Care and nurture. People who have Character, Personality, and Values are spiritually alive. There are seven values of happy healthcare professionals and this includes Invincible exists, Happiness improves our Health, Respect for Being, Ehsas/Benevolence, Righteousness should be evidence based, Equity and Equality besides Consequences of our actions. It is your patients which keep you healthy, Dr. Mowadat Rana remarked.

In their concluding remarks the chairpersons commended the speaker Dr. Mowadat H. Rana for his excellent presentation on how to find happiness. **Dr. Sara** pointed out that this workshop should be for all professionals and they must all learn how to make themselves happy. **Prof. Aftab Asif** remarked that it is very difficult to make all people happy. This prescription of Happiness is different. These issues need to be discussed with the educationists. We all make mistakes. He was of the view that instead of waiting for great happiness let us be content with small happiness and accumulate all these small happiness’s. **Prof. Khalid Mufti** remarked that Prof. Mowadat Rana has covered cognitive, social and spiritual magnet. Good teachers are a must for success. We all need to audit our thoughts, behaviour and avoid doing any harm to the patients.

**Workshop on Children in a Digital World**

Today’s children are ‘digital natives’, who have grown up surrounded by digital information and various screens (TV, Computers, mobiles, tablets to name a few). However, there have been growing concerns of impact of screen time on the health and development of young people. This workshop was organized recognizing the importance of addressing this issue and to increase awareness among Professionals and parents alike.

The aim of the workshop was to provide an overview of the effects of screens on children health and development. Coordinators of the workshop were Dr Nazish Imran from KEMU Pakistan and Prof Waqar Azeem, Chair of Psychiatry / Child Psychiatry for Sidra Medicine in Doha, Qatar. Dr Waqar Azeem is also Professor of Psychiatry at Weill Cornell Medical College / Cornell University, USA. After introduction of topic, the participants were divided into groups with one facilitator each, where they could discuss one aspect of screen time impact on children health and development and gain insight into most current understanding relevant to it. After group work and presentations from group representative, facilitators summarized the evidence base for specific areas of interest.


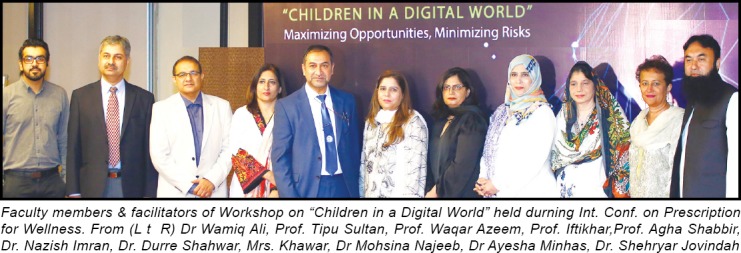


**Dr. Aisha Sanohbar Cha-cha** from Agha Khan University, Karachi discussed adverse effects of screens on Physical Health of the children. Highlighting that media use during preschool years is associated with small but significant increases in BMI, Results of a recent study of 2-year-old children, found that BMI increased for every hour per week of media consumed. **Dr. Vaughan Bell** and colleagues noted that “low levels of physical activity associated with the passive use of digital technology have been linked to obesity and diabetes”. There is some emerging evidence that the devices used to access social media and the Internet may have an effect on the body and its physical development. **Anna Clark** from Cardinus Risk Management highlighted that there was “research looking at backs, spines and posture” and that while the “biological make-up” of children can mean that they “tend not to get repetitive strain as often” as adults, there were ongoing studies examining “children texting with one thumb and texting with two thumbs and how it is impacting on the c-spine”.

**Dr. Ayesha Minhas** highlighted the impact of screen time on child development. Population-based studies, she stated, continue to show associations between excessive screen time in early childhood and cognitive, language, and social/emotional delays, likely secondary to decreases in parent– child interaction and poorer family functioning in households with high media use. Children who started watching television before 12 months and watched more than two hours a day were six times more likely to have language delays. Attention problems are also associated with early screen viewing, with each additional hour of viewing/day at ages one and three years associated with a 9% increase in risk of ADHD diagnosis by age seven. A positive correlation between screen media viewing and Autism based on country data was noted by Waldman et al in 2006.

**Dr. Nazish Imran** (KEMU) and **Dr. Wamiq** ((AKUH) focused on complex associations between screen time and mental health. It was observed that ddifferent types of screen time may have different effects, both in terms of wellbeing and in terms of poor mental health. Furthermore, association between screen time and low well-being was found to be larger for adolescents than in young children. Numerous plausible potential intervening pathways relate young people’s mental health to the amount of time they spend on social networking sites, and the ways in which they engage and interact online. A systematic review of reviews published in BMJ in 2019 found moderately strong evidence for associations between screen time and higher depressive symptoms and poorer quality of life and weak evidence for associations of screen time with anxiety, poorer self-esteem. No or insufficient evidence for an association of screen time with eating disorders or suicidal ideation was observed. Another area that has received attention is the relationship between social media, screen-time and sleep. The young people in studies highlighted how the need to be on social media, and contactable at any time, could disrupt sleep. Furthermore, potential effects of the ‘blue light’ emitted from smart phone and tablet screens on having an effect on a chemical in the brain called melatonin is studied. Melatonin facilitates the onset of sleep.

**Dr. Durre Shahwar,** from CAMHS, Sidra Medicine, Weill Cornell Medical College, Doha, Qatar discussed Psychological aspects of Cyber bullying. Describing the difference between traditional and cyber bullying she noted that traditional bullying end when school ends but with cyber bullying there is no escape. Cyber bullying has a wider audience, affect children in public and private spaces from school to their bedrooms, and escalate in scale quickly due to people sharing or commenting on bullying content. The key elements of cyber bullying involve ‘*boundless space, an infinite audience, unknown bully and low parental presence*’. Highlighting the associational evidence between children’s experiences of cyber bullying and mental health, she gave example of a pan European study which found that 12.2% of victims of cyber bullying had viewed websites associated with suicide compared to 3.7% of people who were not involved in cyberbullying. Focusing on solutions to the issue, she discussed roles and responsibilities of social media companies, to prioritize the promotion of children and young people’s mental health and well-being across their platforms, timely, effective and consistent responses to online bullying alongside parents role as well.

**Dr. Shehryar Jovindah**, from SKMTH Lahore focused on psychosocial conflicts arising in adolescents in Pakistani society context due to excessive exposure to inappropriate content on social media. These psychosocial conflicts in turn leads to various negative outcomes including Agitation, frustration, Anger, Anxiety, Depression and Substance misuse.

Prof. Waqar Azeem and Dr. Nazish Imran mentioned the salient points and recommendations of WHO and AACAP and APA regarding appropriate use of screens by young children. They highlighted that the time limits recommendations for screen time include 0-2 Years: No screen time; 2-5 years: Limit to under 1 hour/ day; Upto 11 years: 2-2 1/2 hrs/ day; and avoiding screens for at least one hour before bedtime. They also emphasized that because parent media use is a strong predictor of child media habits, reducing parental media use and enhancing parent–child interactions may be an important area of behavior change.

Panel of experts in the workshop included Prof. Agha Shabbir, Prof. Tipu Sultan, Paediatricians Prof. Haroon Hamid, Prof. Iftikhar, Dr Mohsina (Child Psychologist) and Mrs Khawar. It was concluded that as digital technologies become more ubiquitous, pediatric providers must guide parents not only on the duration and content of media their child uses, but also on creating unplugged spaces and times in their homes to ensure that we create the good digitalized society where we take advantage of the digitalization and at the same time protect the normal development of our children.

**Perinatal Mental Health**

This session was chaired by Dr. Unaiza Niaz along with Dr. Rukhsana Kausar, Prof.Khalid Mufti, Dr. Sumaira and Dr. Rafia Rashid. The topic of Dr. Unaiza Niaz’s presentation was “Challenges of Urban Working Women’s Mental Health in Pakistan”.

Urban working women, **Dr. Unaiza Niaz** opined have to face too many problems starting with the traffic congestion, long distance travelling, increased social demands in office. They are also faced with self-image issues, fashion and have to keep themselves abreast of global world. She highlighted the issues specially faced by working women in office and in the family. These days, she further stated, we see smart educated patients. Our attire is very important and working women must ensure that they have a modest professional look.

Women, she said, have to work with family conflict, face stress at work and it spills over. Working women are going through stress much more when they comeback from office. Looking after of children is not defined. With job spillover, they face many problems because of long working hours, physical behaviour and they suffer from super women syndrome. They are also supposed to be good housewives. They may be good at work but then working with the family costs in the form of stress. She was of the view that we need to ensure that their stress is handled very carefully, avoid burnout otherwise they lose the raise in their pay. On the whole working women, she opined, are underpaid. Women, who do not work, are not given respect. Wellness for the women, Dr. Unaiza Niaz said, is more important. Women must do some Work, take up teaching as we need good teachers. At times there are questions asked why the women are working? Women who are stressed will not conceive and the gynecologists should know this. She then referred to a study on women physicians which showed high prevalence of depression and suicide. Prevention of these diseases remains relatively ignored. To be suffering from depression is not a weakness. Depression needs to be looked into and treated to avoid burnout.

Royal Colleges in UK, Dr. Unaiza Niaz stated have arrangements where physicians come to seek attention of psychiatrists. All this is kept confidential. We should have this facility in our medical institutions and the problems should be acknowledged. Work place is much better with women if both men and women work together at a work place. Men and women are two sides of the same coin but they think differently. Their dedication to the company will be much higher. Working women love to be recognized as professionals but what happens is that often working women are ignored. Men gossip more than women she alleged. Emotional control, Dr. Unaiza Niaz stated is important and working women should be proud of what they are. Continuing Dr. Unaiza Niaz said that working women love your concern but not your sympathy. At times intimate questions are asked by the employers like marriage, family life. There is more gender bias at working place which is being highlighted these days. She suggested that we must give compliments at what women are doing. We must take care of the women physicians in particular. They must get six months family leave. Institutions need to provide Day Care facilities for the children. She also referred to increasing incidence and prevalence of sexual harassment. Working women physicians suffer from sexual harassment not only from physician colleagues but patients as well. She advised the women physicians to have proper dress while sitting in the OPD and at work place. They must look like a Doctor. Your attire will make you good. Avoid high neck, sleeve less shirts. Deans and VCs of medical institutions should help women to carry on their career. They should get twelve weeks paid family leave and four to twelve weeks child rearing leave. There should be arrangements for on-site child care services. She also supported the concept of career flexibility for women, improvement in mentorship, sponsorship. She concluded her presentation by advising the working women to choose their friends carefully.


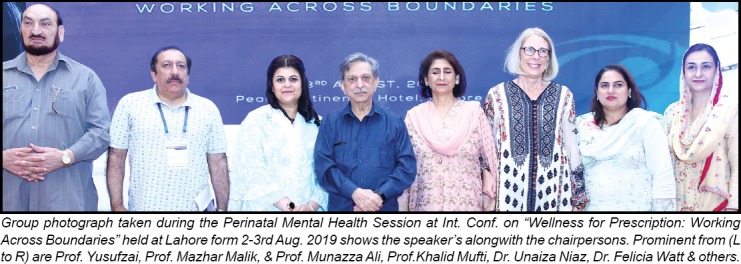


**Key issues in Women’s Mental Health**

**Dr. Felicia Watt** from Qatar highlighted the key issues in Women’s Mental Health. She pointed out that some disorders are gender specific. She emphasized the importance of relieving the symptoms, taking care of the known risk factors. There is increased exposure of women to violence, some attempt suicide. Depression, anxiety, Post Stress Traumatic Disorders are quite common. Women have low income, in some countries women are not living with their partners but alone. There is lack of emotions, some women suffer from low level of education, many of them remain unemployed which results in financial strains, and they have greater exposure to poverty. Substance abuse is twice more common in women and there is increased exposure of women to stress as well.

**Psychopharmacological considerations in pregnancy and lactation**

**Dr. Syeda Munazza Ali** from Qatar discussed psycho pharmacological considerations in pregnancy and lactation. She pointed out that those women who are contemplating pregnancy or already pregnant should consider risk and benefit of taking prescribed medications. These are some of the challenges which women physicians are facing. Pregnant women have several mental health issues with current medication besides non-prescription drugs they are taking. There are issues related with weight management, exercise supplements. No medication, she remarked is safe in pregnancy. Drugs do pass through placenta hence we have to weigh the risk and benefit to women as well as her fetus. We must find out what effects the drugs will have on the fetus if the women are treated and if not treated? Some pregnant women may have late preterm labour, it could also result in low birth weight babies. Up to sixteen weeks of gestation period is high risk for congenital malformations and studies have showed that drugs are responsible for 4% of congenital malformations. Radiation during pregnancy has its own side effects, she remarked.

Continuing Dr. Munazza Ali suggested that while treating pregnant women, the treating physicians must talk to the women and their husband. It is important to involve obstetricians and neonatologists while treating pregnant women suffering from mental disorders. To find out the teratogenicity effects of drugs, women are not part of the research as RCTs are not possible. It is difficult to find out side effects of drugs from behaviour consequences. Depression, smoking, lack of folate, use of alcohol all have their effects during pregnancy. Then there are some physiological changes during pregnancy. Physicians must be aware of general principles of drug prescribing during pregnancy. She stressed the importance of engagement with the patient, collaboration during treatment and avoid switching over of drugs as far as possible. It is also important to avoid the use of high dose of drugs during pregnancy, ensure exclusive breast feeding during the first six months. Psychotropic drugs do pass into the breast milk and these issues needs to be discussed with the patient taking into account the risk and the benefits. Anti-depressant can be prescribed during pregnancy and when used in low dose, they are safe during breast feeding. It is essential that we consider the gestational age as well as co-morbid conditions of the patients.

She emphasized the importance of monitoring the baby for adverse effects. Pregnant women are a special population, they require different dosage regimen. Change in drug metabolism is observed during pregnancy and fetal exposure to different drugs varies, she remarked.

**Wellness across the Life Span**

The session was jointly chaired by Prof. Waqar Azeem, Prof. Mowadat H. Rana, Dr. Rubina Aslam, Dr. Ajmal Kazmi and Dr. Moammad from UK. Prof. Aftab Asif, Head of the Dept. of Psychiatry at KEMU talked about Transcranial Magnetic Stimulation (TMS).

**Prof. Aftab Asif** pointed out that there are different options to improve mood of the patients. Various modalities were available in the past. Transcranial Magnetic Stimulation is a non-invasive procedure. Through this electric current is induced in that depolarized neuron in focal area of cortex or deeper brain structures. The whole procedure lasts for more than six months. It is effective in many resistant diseases. It was generated after fMRI. Brain, he said, has different areas involved with different emotions. When the antidepressants, SSRIs and psychotherapy fail, they start using TMS. This treatment is effective and it was approved by FDA of United States in 1984-85. It was first developed by Anthony Barker and is known as brain stimulator. Currently it is being used for major resistant depression, Obsessive Compulsive Disorders (OCD), Anxiety disorders including PSTD. It is also being used for research in brain mapping. Some of the countries where it is being used successfully with excellent results include USA, Canada, New Zealand, Australia, and European Union.

Continuing Prof. Aftab Asif said that FDA approved it for resistant depression in 2008, for migraine headache in 2013. TMS is advocated by stimulating and it is a focused treatment. The procedure has to be done daily in male as well as females for three minutes. The treatment is three thousand pulses per session. In acute cases treatment lasts for four to six weeks and it does take time to show results just like antidepressants or psychotherapy. Brain activity is detected by changes associated with blood flow. This TMS treatment can be combined with other treatment modalities like EEG. It uses functional MRI and is more effective in resistant depression. Speaking about its benefits Prof. Aftab Asif mentioned that it is an OPD procedure. There is no need for anesthesia; the whole procedure lasts for three to four minutes. During the first visit of the patients, it may take forty five minutes. There is no memory deficit. It is safe and well tolerated. Some of the adverse effects include headache scalp pain or generalized seizures which are 0.2 to 0.5%.

**Prof. Naim Siddiqui** from SIUT Karachi discussed different ingredients for wellness. These ingredients, he said, are overlooked or under estimated. Studies have shown that train or aircraft nose impair cognitive development of children. These children have increased hyperactivity, lower level of physical activity. Traffic noise is also related to development of hypertension. He also referred to the Railway and Aircraft noise and their related physical side effects. He discussed in detail the noise pollution and its related physical consequences. Referring to the Air Quality Index, he said that traffic noise and air pollution are risk factors for hypertension and diabetes mellitus. Greenness is protective. Air pollution is related to many diseases. Studies have showed that life style changes improve health by 58% compared to Metformin which shows positive effects in 31%. Life Style Changes delay the development of diabetes for three years. It also leads to reduction in weight coupled with sensible choice in diet. But we have too many excuses not to change and poverty is one such excuse. However, it is not the poverty alone but traditional sweets as we do not use fruits. We are used to high intake of salt, refined food and junk food. He also referred to meal size and the frequency of food. Children suffer from learning disability. If the child is not healthy, we cannot think of wellness. It is essential that the mothers should have appropriate diet. Let us accept what cannot be changed. It is the courage to change which can be used to distinguish between others. One Banana, few Almonds, one cucumber, and a lemon is what we need daily to remain physically active in adult life. It is the health beliefs which matter and it is not still too late to practice this, Prof. Naim Siddiqui remarked.

**Prof. Farid Aslam Minhas** from Rawalpindi discussed the Borderline Personality Disorders and the birth of different interest groups in Paksitan. He started his presentation by paying rich tributes to late **Prof. Ijaz Haider** and late **Prof. Mohammad Sharif** the two renowned psychiatrists who passed away few months ago. They were the pioneers in the field of psychiatry and both of them have contributed a great deal to the promotion of psychiatry and mental healthcare in Pakistan, he added.

He then discussed in detail the assessment and management of borderline personality disorders, assessment and management of cognitive dysfunctional depression in old age and the parental guidance- prevention of childhood traumas. He disclosed that they have formed these interest groups. Some of these disorders are difficult to treat and manage. They require personalized management. Childhood trauma patients have serial physical abuse. Most of them have suffered abuse in their early life and it inflicts more damage. Children who are well looked after by the parents have good effect. Some of these patients have mix of intense emotions regarding self harm and suicide. They also suffer from other co-morbid disorders like depression, anxiety disorders, sleep disorders, substance abuse. It is a long term chronic disorder. The period of stress is much longer.

Speaking about the goals of treatment, Prof. Farid Aslam Minhas mentioned emotional regulation, muscles and emotional reactivity. They may not be fully cured but it can be controlled. There is need to secure bond with mental healthcare professionals. Borderline Personality Disorders, he said, are here to stay and let us start talking about it without wasting any further time. However, it is extremely important that mental healthcare professionals should know how to say, what to say and how much to say. Unfortunately some of the professionals lack knowledge, skills and have no time to effectively address patient’s needs. The therapists and families of the patients also need support.

In their concluding remarks **Dr. Ajmal Kazmi** said that TMS is an expensive treatment modality but it gives better results in carefully selected cases. It is heartening to note that this facility has been made available by Prof. Aftab Asif in Lahore.

**Prof. Waqar Azeem** referred to the issues related to the off label use of different treatment modalities and the legal issues involved. That is why it cannot be prescribed for emotional disturbances. To do that, we need data and evidence, till then we cannot use. He suggested that the local data should be compiled and its results should be presented at the next conference. It is important that the therapeutic efficacy of all new treatment modalities, procedures are presented at professional forums. We all remember what happened to Prozac which later became known as the “Happy Pill”. Interventions like TMS are for serious disorders. It should be used with caution and not misused like ECT. Referring to Prof. Naim Siddiqui’s presentation, Prof. Waqar Azeem said that we all know what is good for us but we do not eat it. Prof. Farid Aslam Minhas dealt at length on assessment and management of borderline personality disorders which is a reality and we need to know how to effectively manage it.

**The Psych Lounge**

One of the most important sessions during the Conference was The Psyche Lounge. This session was chaired by Prof. Waqar Azeem a renowned Child Psychiatrist along with Dr. Amina Ahmed a medical educationist, Dr. Saida Saleem, Dr. Tazvin Ijaz, Dr. Durre Shahwar, Dr.Ali Hashmi and Dr. Nazish Imran. This session was moderated by two brilliant medical students Mr. Salar Haider and Suchna Meeral Khan. During the whole deliberations some exceptional contributions were made to the discussion by Dr. Ali Hashmi, Dr. Amina Ahmed and Prof. Waqar Azeem, Dr. Tazvin Ijaz not to forget the other panelists and experts who also enriched the discussion with the valuable comments.

Some of the important questions raised in the beginning of the session were the reasons and major stressors for growing mental health problems among the students in our colleges, the stigma, what are the barriers to reaching out and seeking help and how does one identifies the red flag in one’s self and others and how do we seek help besides how mental illnesses are treated and what was the role of medications? These are all very important questions indeed. It was pointed out that it was extremely important to identify the mental health issues as early as possible. However, people start neglecting it. We must know what interventions these people need and must also keep in mind the stigma in public and among the healthcare professionals as well. The role of faith healers also figured up during the discussion. It was highlighted that when something unusual is happening, we get offended. We need to accept the problem exists, only then one will seek treatment. At times it is late. Sometimes the patients do not have good experience with the doctors. We need to help patients to overcome all this as early intervention ensures better and good outcome. It is also important that services should be available and we must pick up these patients with mental disorders as early as possible. We have scarce resources, very few qualified trained psychiatrists, and hence these services are not easily available all over the country. There are numerous barriers to seeking help. Psychiatrists are not available in small cities and towns hence it is essential that mental health resources are made available.

While discussing the role of medications, it was stated we must discuss is it enough? This, some of the panelists opined, was a unique session during the conference and your wellbeing is important but primary prevention is all the more important. For any intervention, scientific evidence should be there regarding its safety and efficacy. Anxiety and depression are most common in medical students. It is more common during the first two years particularly among those who live in hostels. These disorders are also more common in female students. Since we have less time we think medication is the solution although social factors are more important than biological. We need supportive teachers, medication is the last. Psychological interventions should be preferred and medication is only needed when all functions are severally affected. **Dr. Nazish Imran** stated that student’s health centers should be set up at all medical institutions. There are institutions which have three to four full time counselors on the campus. These health centers should look after health of the students including mental health. They should be doing better. At times there is reluctance to see psychiatrists hence it is better that these health centers are located in the university rather than in the hospital which will be an added barrier.

There are students, teacher’s dilemma due to communication gap. It is essential that the teachers should come down to the level of students. It is the teachers who should take the lead and open communication channels with the students. They should encourage the students to open up and discuss their problems and talk about their issues. The teachers should not be judgmental. They must come to the same level. **Dr. Ali Hashmi** opined that we teachers also learn from the students. At times some students are treated unfairly. Dr. Ali Hashmi highlighted the importance of accountability of the teachers and felt that the students should assess their teachers. The King Edward Medical University, he further stated, has such an assessment Form on its website. He encouraged the students to give their feedback about the teachers. It is not only the undergraduates but postgraduates as well who should give us the feedback. Teaching modality should be changed. It was pointed out that the confidentiality of those who assess the teachers and give their feedback should be ensured. It was important to change the institutional culture to get honest feedback. **Dr. Nazish Imran** stated that all teaches should be assessed. The university authorities, administration can also post some people to assess the teachers. There is a generation gap between the students and teachers which affects the relationship between them. **Prof. Waqar Azeem** was of the view that the teachers should be trained how to identify the problems. They must talk to the students affectionately. Teachers should be considerate, accommodating and supportive. They should help students to cope with mental health issues. Teachers, he further stated, are part of the system and we need to fix the system. Lectures are good and bad and we need to find out why they are so. We do teach the students good medical knowledge but social, personal leadership skills are neglected. We need to make them good human beings which will make them better doctors. Students will follow the teachers. Teachers, **Dr.Ali Hashmi** stated, must be able to say we do not know the answer. Say sorry if something is wrong. They need to fill up the gaps between parents and friends. They should help and guide the students. Recreate things with more light; ensure help by students and teachers. We need to improve the curriculum to attract students. Students are not interested in lectures but they need more discussions.


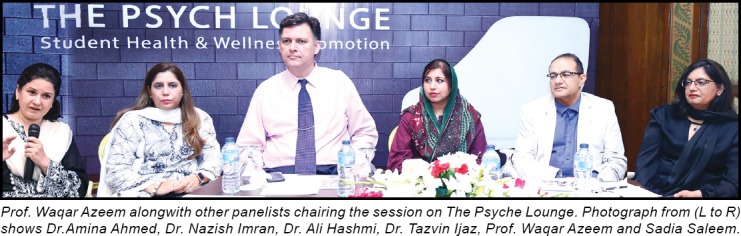


The issue of mandatory attendance also came up under discussion and it was pointed out that it is the requirement of the regulatory bodies; hence we have to maintain it. Though we know at times students mark attendance for others not present in the class as well. **Dr. Amina Ahmad** remarked that teachers should be engaging. According to the principles of adult learning, students will pay more attention if the teaches are more passionate about teaching. Particularly in basic sciences people do not come by choice. It was also pointed out small group teaching is much better and more effective but in large group of students this does not work. We need much more resources, faculty and other teaching training facilities for small group interactive discussions. One does not become a teacher just after getting some postgraduate degrees. Some degree or diploma in teaching is essential. It is good that now the PM&DC has made it mandatory that all faculty members must have at least attended a Certificate Course in Teaching, which has been started by many medical universities and other medical institutions. **Dr. Tazvin Ijaz** pointed out that at Government College, they have student’s health center at the institution and they have full time counselors. We look at the students problems. We need qualified people to work as counselors, she further stated. **Dr. Amina Ahmed** opined that intervention in the home institution is the good option. Now research has assumed much more importance and students are encouraged to undertake research and they get due credit and points for this when they apply for jobs or Residency in UK, USA, if they have done some research before graduation. Self-directed learning is immensely important

One of the participants referred to high suicide rates among medical students in one of the studies. Responding to this **Dr. Ali Hashmi** said that there has been no such study on this as far as he knows in Pakistan. Role of good mentors was also highlighted. It was also stated that some medical teachers are in the medical institutions for other reasons but they have no passion for teaching. Every institution, it was said, should have an internship and research programme. At this one of the participants complained about the red tape in getting hospital data for her research topic from various hospitals. **Dr. Amina Ahmed** responded by saying that there are some ethical issues involved and these restrictions are in place even in UK and USA. Some identity and information has to be protected but as regards the fear that the topic of your research will be leaked out to others, it is the duty of the hospital to keep all the documents submitted by the researchers confidential.

Dr. Amina Ahmed further stated that we also need to modify our examination system. Assessment should be valid and reliable. It is important to know how the attitudes are being taught. What quality assurance measures are put in place? Curriculum and examination system needs major overhaul. It is said that the First and the Final professional examinations are the killers. Assessment should be done during the whole year. One can have just Pass and Fail and not bother about the Grades. We must inculcate the concept of thinking in the students. We need to do problem solving. Questions should force the students to think. Students should be represented in the Academic Councils though at present it is being resented and opposed by some senior teachers. Dr. Amina Ahmad remarked that in the recent PM&DC regulations the students are supposed to be in every committee. **Dr.Ali Hashmi** opined that in the past representatives of students unions used to perform this duty. We need to get back to that minus the politics in which students unions used to indulge. These students’ representatives in different committees should be selected by the students themselves.

During the discussion it was also stated that the Third and Fourth year students can help and guide the First and Second year students. Many professors have a paternalistic attitude and approach as in our society, it is the parents which most often decide what is good for their children, including their choice for further studies, professional career and even when and where they will get married. Dr. Ali Hashmi was of the view that the children should be given the choice to explore their own passions. There are instances where parents force their children to become doctors. Passionate students will be much better doctors than those who opted this profession under compulsion. Government College Lahore it was stated has qualified people as counselors and mentors. These counselors should also be held accountable as to what they are doing for the students. Dr.Ali Hashmi suggested that before getting admission to medical colleges, the students must go through medical, physician, mental and surgical checkup.

Summing up the discussion **Prof. Waqar Azeem** commended the panelists as well as the participant for their excellent thoughts, suggestions. He particularly commended the students who had moderated the session saying that he has never seen such a good session at any international psychiatric conference. Credit for all this of course also goes to the organizers **Prof. Imran Ijaz Haider** and **Dr. Nazish Imran** for having thought of such innovative ideas and introduced many innovative things at the conference.

**Dr. Durre Shahwar** was the first speaker in the next and she discussed Management of Eating disorders in adolescents. She pointed out that abnormality in eating pattern is seen during individual’s attitude towards weight and shape. She discussed at length Anorexia, Bulimia Nervosa and atypical disorders. As per ICD-10 criteria, anorexia is body image distortion, dread of fatness, imposing low weight threshold. It leads to endocrine changes of amenorrhoea in females. Bulimia nervosa is recurrent episodes of overeating.

He then discussed in detail a self-critical approach analyzing body shape and weight, Anxiety and low confidence, restoration of food intake and the psychological factors. He also referred to family lack of conflict resolution. Malabsorption, chronic infections, illicit substances, prescribed medications were mentioned in differential diagnosis. Teen agers, she said, may start simple dieting. Some may lose control. Diagnosis is based on initial face assessment. Speaking about signs and symptoms she mentioned dramatic weight loss. She advocated family based approach for management of these patients. Assess eating habits, return control over eating to adolescents. Psychological therapies and family therapy is useful empowering the parents. Create awareness about problems of eating disorders in which media can play a very important role. It has its impact on mental and physical health. Media often highlights the images of underweight celebrities, she added.

**Prof. Shazia Maqbool** from Children Hospital Lahore talked about autism disorders. Its prevalence, she stated is between 0.2-5%. It has four times increase in nineteen years and studies have reported 15-19% recurrence in siblings. She pointed out that more children suffer from Autism Spectrum Disorders than diabetes mellitus and Aids or Cancer. Now there is more awareness about autism disorders. She pointed out that anti-social behaviour may not be autism. These children are non-communicative. Adults are role models. Children live is isolation if their parents are active on social media. Though these people have too many friends on social media but when they die, one does not see many people attending their Nimaze Jinaza, she remarked.

Continuing Prof. Shazia Maqbool said that the average age of diagnosis of Autism Spectrum Disorders is about 4.5 years. Impairments are observed at twelve to eighteen months. She advised the parents not to wait. Pediatricians are now much more educated to suspect ASD early in life. These children do not have an eye contact at four to six months hence impairment should be looked at. Those children who are not responding by fourteen months must be immediately referred to specialists. Six months child, she said, should look at the object which is pointed out. Avoid mix up of diagnosis i.e. Behaviour communication disorders, social communication disorders, language disorders. Find out if there is no eye contact; look at the hand movements, and use of objects. She then showed a video of sensory deficit, frustration, and anger and highlighted the importance of one window diagnosis. She then described in detail the one window model which consists of multidisciplinary services which consists of fetal medicine, educationist, psychiatrist and nurses. History is extremely important. Parental report, examination, developmental profile, generalized and specific investigations, speech assessment, child behaviour inventory are all important. She suggested multi professional approach including audiologist. NGOs have a great role to play to pay attention to family distress. We at the Children Hospital, Prof. Shazia Maqbool stated, have established a full- fledged department of developmental pediatrics. Early intervention in autism ensures good results. Parents support is very important and they need to become our partners. As regards drug therapy, antipsychotic Resperidon is approved by FDA. Treatment is for symptoms and not the disease. We have established a learning center. These kids can be kept in schools and some of these centers are our partners. In the OPD almost 33% of the patients we see are of cerebral palsy. She concluded her presentation by stating that winners never quit.

**Madam Rukhsana** talked about shared care for care givers. She highlighted the emotional and financial burden of care givers. They have to look after the needs and disabilities. They are supposed to have a positive attitude. Diseases like autism, ADHD, ODD, OCD have a heavy toll on parents. Almost 80% parents of autism children have stress. She also referred to the low level of social support available. Afflicted parents, care givers need proper counseling. They also need training, support from the government and society. She then showed a video of autism. The child never spoke a single word. She then referred to Indian approach and gave details of Action for Autism. The Autism Center is located at ten acres of land.

Speaking about solution to the problem she mentioned trained doctors, teachers, and therapists. All medical colleges and universities should have a department of psychology. Autism should be included in their curriculum. Autism centers should be established in all big cities. They should be provided funding for training, support for rehabilitation and independent living.

**Qatar National Autism Plan**

**Prof. Waqar Azeem** highlighted the excerpts from Qatar National Autism Plan and they are one of the four countries to have a National Plan. Pakistan, he opined, also need to have such a national plan for autism. In 2019, Qatar has about 2.8 million populations. We had a working plan from 2013-2018. Lot of infrastructure is being developed. Government is spending lot of funds on Health and Education. We have formed various working groups. Previously we had no data, hence no services were available. We faced problem in diagnosis hence we formed Six Task Forces which are working in the field of awareness, education. The main task force is headed by the Prime Minister; six ministers are its members which includes the Health and Education ministers. We have a five years strategy and during the last two years we have made some progress. We have nine clinical psychologists, three perinatal psychologists and we have an excellent facility in Qatar he added. He also showed the pictures of various stages of development of Qatar and at present the country has a large number of high rise buildings which have all given Qatar a new look as one of most developed country in the region, he added.

**Prof. Riaz Bhatti** and **Dr. Saeed Farooq** made presentations through Skype from UK. Prof.Riaz Bhatti talked about looking after the families of mentally ill patients while Dr. Saeed Farooq discussed Treatment Response- Remission and Resistance in psychosis. He discussed in detail the obstacles in achieving remission and treatment goals in schizophrenia. It also leads to remission and then full recovery which means autonomy. Response and maintenance leads to suppression of stress and stability while remission is important in social functioning. He then referred to clinical and global impression scale for schizophrenia. He laid emphasis on optimal use of pharmacological interventions, consider switching over, response to antipsychotics should be carefully measured. He also talked about psychosocial interventions besides the use of clozapine. Only about 30% of patients who are eligible to take it get this drug. One must consider switching over if a particular drug is not working. Speaking about the side effects of antipsychotics he mentioned cardiomyopathies, epileptic seizures, agranulocytosis and metabolic syndrome.

** Dr. Omar Mehmood** from Qatar was the first speaker in the last scientific session who talked about maintaining psychological wellbeing and resilience: strategies for therapies and caregivers working with emotionally demanding patients. He highlighted the importance of training young psychologists. Burn out is expected because they see so many chronic cases. Caregivers or parents of these children have numerous problems. Since they focus on child wellbeing, they ignore their own mental health. He emphasized the importance of maintaining the psychosocial health and self-care of these caregivers. If they look after their own health, they will be in a much better position to look after the loved ones.

Continuing Dr. Mahmoud said that these care givers must take a break. Come back more relaxed to look after their children. As therapists we know life style changes, holiday and break are good for self-care. They have no dedicated time. People working on the front line have no time for their self-care. They should have a dedicated time to look after their own mental and physical health. Work load should be equally distributed among all members of the healthcare team. Some people do good work in difficult situations. Paediatric oncology is a very difficult field to work in. You must know your personality and how best you can take care. He further stated that the role of spirituality is very important in self-care. It is getting popular and getting more importance. It also offers a great advantage to Muslim therapists. He also talked about stress reduction techniques. Drugs will benefit therapists to reduce stress, he added.

**Dr. Mohammad Al Rawi** from UK discussed care giving for patients with addiction. His presentation was based on a case history of a thirty years old female emotionally unstable. She was suffering from personality disorders, risky behaviour, indulged in over spending. She had huge debt spending family credit. In aging population family members have to take care of complex care tasks. It results in great cost to their own mental health. About 19% suffer from both mental and physical disabilities. Almost 49% of women who suffer are between 45-64 years of age. In one of the surveys about 30% rated their health as fair or poor while 49% had long standing health problems. Caring responsibility made them feel worried. About 32% said they feel depressed, suffer from stress and tension. He also talked about social and cultural factors and highlighted the importance of Carer’s Support for Groups training and counseling. Carer’s assessment is also important, he remarked.

In his concluding remarks **Prof. Naim Siddiqui** said that clozapine was indiscriminately used in developing countries. Dr. Mohammad Omer has highlighted the importance of giving time for our self-care. Spiritual bondage is important. One should relax. Dr. Mohammad has given us an insight in UK practice of care giving. In Pakistan it is the family members who take care of such people while it is the professionals who take care of such patients in the West. **Prof. Azizur Rehman Yusufzai** said that we must take care of our sick old parents. It is good and Swab. Home based care is important. He suggested Prof. Waqar Azeem to provide the copies of the Qatar National Action Plan for Autism so that we in Pakistan can also benefit from it. It is said that behind every successful man there is a women but I would add that behind every successful women, there is a man and we have seen it during this conference, he remarked.

**Rehabilitation and Social Psychiatry Session**

This session was chaired by Prof. Mazhar Malik along with Dr. Humaira Saeed and Dr. Attiya Inam. **Dr. Ajmal Kazmi** from Karachi was the first speaker who talked about rehabilitation practices. Psychosocial rehabilitation, he opined, is a multidisciplinary teamwork but this approach was lacking in Pakistan in the past. Now numerous developments have taken place and the situation was much better. Every patient, he further stated, is an individual and what level he or she can achieve is also different. Treatment must be tailor made for individual patient. We need to work how the patients can cope with their illness, eliminate their functional deficit. We need to overcome eventual barriers and create new progress. Treatment modalities consists of combination of science and art which ensures patients recovery. He also talked about community integration, improvement of quality of life of the patients and the goals of psychiatric rehabilitation. We need to involve the patient in their treatment, assess their need for a change, create emotional and self-awareness. Closeness to practice has more chances of recovery of the patients. Rehabilitation diagnosis is based on rehabilitation goals. He laid emphasis on evidence based practice, supported employment, proper medication management, personal recovery and progress. All this requires four to eight weeks session with trained practitioners. We must inspire people to become hopeful. Different modules are covered in different courses. He also talked about the teaching strategies, listen to the patient, and motivate the patients. Patients not only need sympathy but empathy was much more important. We must ensure the patients that we know and understand their problems.

**Prof. Mazhar Malik** in his presentation spoke about social psychiatry as a national movement which can change our psychiatric practice. He highlighted that goal of effective treatment should be enabling holistic recovery and not mere reemission of the active symptoms.


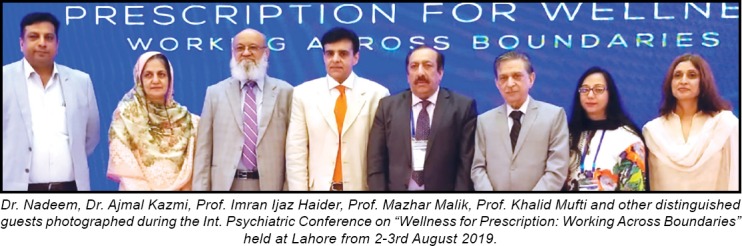


**Prof. Zahid Mehmood** focused on wellness as a goal of health. **Prof. Mamoona Mushtaq** talked about psychosocial factors of addiction and impediments after rehabilitation. **Prof. Khalid Mufti** discussed the importance of spirituality and its role in mental healthcare. **Prof. Shahida Batool** spoke about the caregivers’ issues in dealing with patients of dementia. Health, she stated, in its true sense was moving towards prevention rather than treatment. It is extremely important that brighter minds should come together at such conferences and create awareness about the importance of mental health and promote those institutions which are working in finding solutions to our problems in this field.

**Concluding Session**

**Prof. Javed Akram** Vice Chancellor of University of Health Sciences was the chief guest in the concluding session. He suggested that a Task Force on Psychiatry should be formed so that it gives suggestions to improve mental healthcare by making appropriate changes in the undergraduate and postgraduate medical curriculum.

Continuing Prof. Javed Akram said that we all need to be well so that we can look after the patient’s well which will ensure their health as well as happiness. Majority of the patients suffer from some sort of mental disorders. Depression and anxiety are very common hence it is important that every doctor should know how to manage these patients and treat depression, anxiety and also know when and to whom the patients should be referred for further work up and specialist care if need be. He suggested Prof. Imran Ijaz Haider Chairman of the Organizing Committee of the conference to form the Task Force by inviting mental healthcare professionals who can contribute and come up with the suggestions so that appropriate changes in the curriculum can be initiated.


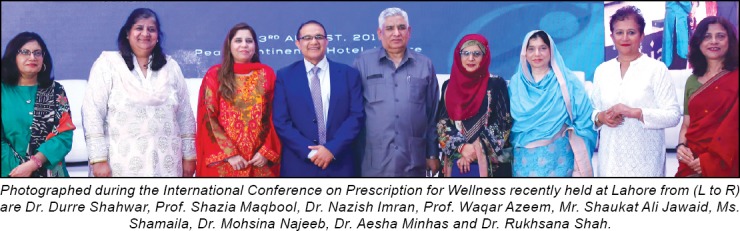


He further stated that we cannot do much about genetics but we can effectively handle stress and strains of life. It is also a fact that stress improves performance but if stress persists for long, it can be dangerous, hence it is advisable not to be over stressed. We all must know how to work under stress and it is up to the experts and Peers to guide the healthcare professionals on these issues. We being Muslims have full faith in Holy Quran and there are many things which we cannot change in life. Hence, why to take stress and why regret, he asked? Meditations, prayers, Zikar are all helpful. When we do Zikar, we forget all other things and studies have showed that it reduces the systolic blood pressure by 25% and diastolic blood pressure by 7%. Heart rate also improves but it is important that one must believe in these things.

Prof. Javed Akram further added that it has been proved that post prayer blood pressure lowering affect remains for two and a half hours. Counseling for myocardial infarction patients results in better reduction in heart rate. It is also well known that almost 50% of women suffer from depression. Hence we need to work on prevention of these disorders. It is up to the psychiatrists to guide us so that we can reduce the worries of housewives as well as working women. Healthy women will ensure healthy kids. Almost 50% of stress is work related which can be prevented. We cannot have good physicians unless they also know some basic psychiatry as it is an essential component of medicine. We all must know where the physicians and GPs should refer the patients suffering from serious mental health disorders, he added.

Earlier **Prof. Saulat Siddique** Associate Dean Clinical Affairs at Fatima Memorial Hospital who was the Guest of Honour in the concluding session also briefly addressed the meeting. He commended the innovations in the scientific programme.

Earlier during the day a special session was devoted to pay tributes to two leading psychiatrists **Prof. Ijaz Haider** and **Prof. M. Sharif** who passed away during the last few months. A number of speakers recalled their services to promote the discipline of psychiatry and improve mental health in the country. This session which was named “You Made Us” was moderated by **Prof. Imran Ijaz Haider** son of Prof. Ijaz Haider and himself now a renowned psychiatrist.

